# Healthcare Utilization Unchanged in the Control Arm of a Randomized Clinical Trial

**DOI:** 10.1177/21501319251379740

**Published:** 2025-09-27

**Authors:** Pratik Gongloor, Saad Nadeem, Xiaoying Yu, Mukaila Raji, Kristina D. Mena, Elizabeth M. Vaughan

**Affiliations:** 1 University of Texas Medical Branch, Galveston, TX, US; 2 Texas A&M School of Medicine, Bryan, TX, US; 3 University of Texas School of Public Health, El Paso, TX, US; 4 Baylor College of Medicine, Houston, TX, US

**Keywords:** resource-limited or low-income, Hispanic, diabetes, Hawthorne effect, control, clinical trial

## Abstract

**Background:**

In low-income settings, clinical trial participation may influence participant behavior, including among control groups. Increased access to care and heightened health awareness during trial enrollment could lead to altered behaviors, a phenomenon known as the Hawthorne effect, which may obscure true intervention impacts; however, this effect remains poorly studied in low-income environments.

**Aim:**

To conduct a secondary exploratory analysis of healthcare utilization among control participants of a randomized clinical trial (RCT).

**Methods:**

We retrospectively analyzed electronic medical records from the control arm (n = 26) of an RCT involving low-income Hispanic adults with type 2 diabetes receiving care at a community clinic. Before randomization to a 12-month diabetes education intervention or usual care (control), participants underwent on-site measurements of HbA1c, blood pressure, and weight. Healthcare utilization among control participants was compared during the year before and throughout the study, including all types of exposures: provider visits and other services (eg, orders).

**Results:**

Total healthcare utilization was similar between the pre-period (11.9 exposures/year) and the study-period (11.4 exposures/year; *P* = .93), with no significant changes across visit types. There were no significant differences in fitted mean monthly visits between the pre- and study-periods (*P* = .93), nor over time (*P* = .89).

**Conclusions:**

This exploratory study found no evidence of a Hawthorne effect on healthcare utilization among control participants. While this may suggest consistent healthcare behaviors, it may also highlight an important public health concern: individuals in low-income settings may lack the resources to translate increased awareness into health-related action. Larger studies are needed to further elucidate behavioral patterns in low-income populations.

## Introduction

In low-income healthcare settings, participation in clinical trials can influence patient behavior, including among control group members. The Hawthorne effect refers to behavioral changes that occur when individuals are aware they are being observed or studied.^1^ First identified in the 1920s, this phenomenon has been widely examined across disciplines such as psychology, sociology, and healthcare.^1^ Its impact on healthcare research is particularly important, as awareness of observation can influence study outcomes.^2^ Participants may improve protocol adherence, make healthier choices, or alter their behaviors and self-reports simply because they know they are being studied.^3-6^ This effect can lead to either underestimation or overestimation of an intervention’s true impact, potentially compromising the validity of research findings.^6-9^

Despite widespread recognition of the Hawthorne effect, significant gaps remain in understanding this phenomenon. The original Hawthorne studies were limited by methodological weaknesses, including the absence of a control group and failure to account for confounding variables.^10^ Recent research has struggled to consistently quantify the Hawthorne effect, hindered by heterogeneity in study populations and designs, as well as differences in the nature and frequency of observations intended to elicit the effect.^2,11,12^ These challenges have led some investigators to suggest that observed behavioral changes may stem from external factors such as experimental bias, context-dependent influences, or methodological flaws, rather than a genuine Hawthorne effect.^2,3,12^ A systematic review of 14 studies reported a minimal Hawthorne effect (odds ratio [OR] = 1.17; 95% confidence interval [CI] = 1.06–1.30), but heterogeneity limited conclusions about its mechanisms and conditions.^2^ Another review reported a stronger effect (OR = 1.41; 95% CI = 1.13–1.75); however, the authors noted that the effect was absent in well-designed studies and cautioned that it may overlap with placebo effects and regression to the mean.^12^

The Hawthorne effect remains underexplored within low-income populations, where chronic disease prevalence is highest and the influence of socioeconomic factors is not well understood. It is possible that vulnerable groups may exhibit either increased or diminished susceptibility to this effect. Systemic barriers—including limited access to healthcare, language challenges, and economic stressors—profoundly shape health behaviors in these populations, potentially exerting a stronger influence than study participation itself.^13-15^ These barriers may drive increased clinical encounters among control participants, thereby obscuring the true impact of interventions.

To better understand potential behavioral changes within the control arm of clinical trials, this study explored healthcare utilization among control participants in a randomized clinical trial (RCT) conducted at a nonprofit community clinic. Participants were randomized to receive either a 12-month, multidimensional diabetes education intervention or usual care (control).^16^ This analysis focused on evaluating changes in healthcare utilization among control participants following receipt of baseline clinical measurements, including HbA1c. Specifically, we compared clinical encounters during the year before the study (pre-period, September 2021 to August 2022) to those during the study period (study-period, September 2022 to August 2023). We hypothesized that awareness of being observed and receiving baseline health information would lead to increased healthcare utilization, consistent with the Hawthorne effect.

## Methods

### Study Design and Setting

This cohort study is a secondary analysis of a 12-month RCT conducted at a non-federally funded, nonprofit community clinic in Greater Houston, Texas, serving low-income populations.^16^ To qualify for clinic services, patients were required to be uninsured and have an annual income of ≤150% of the federal poverty level. More than 50% of the clinic’s patients were undocumented. The study was approved by our institutional review board, and all participants provided informed consent.

### Participants

Potential participants were identified through a database by coding for the following inclusion criteria: Hispanic/Latino(a), type 2 diabetes (ICD-10 E11.X), and adult (≥18 years). During the consent process, research staff confirmed that individuals spoke Spanish. We excluded individuals who did not attend any clinic appointments during the 12-month study, were pregnant during the pre-period and/or study-period, or had conditions that may alter HbA1c levels, such as intermittent steroid use.

### Intervention

The intervention was described in the primary study.^16^ Briefly, individuals were randomized to a 12-month multidimensional diabetes program, called TIME (Telehealth-supported, Integrated Community Health Workers (CHWs), Medication Access, and group Education), as the intervention group, or to usual care in the clinic as the control group. Usual care consisted of routine provider encounters for chronic disease management, typically scheduled monthly to semi-annually depending on HbA1c control. Usual care also included opportunities for food assistance, nutrition education, children’s programs, and pastoral care. Intervention participants received communication from CHWs through text and phone calls 2 to 4 times per month, monthly diabetes education videos created by the research team on YouTube, and 8 group visits that included CHW-led diabetes education and provider encounters for disease management.

### Measures

We gathered monthly clinic usage data for the control arm for the pre-period and the study-period through chart review in the electronic medical record. We included the total number of visits per month and categorized them into the following visit types: provider (MD, DO, or Advanced Practice Provider), nurse (eg, blood pressure checks), medication (eg, refills), and other (eg, lab orders and eligibility paperwork).

### Statistical Analyses

All analyses were performed using SAS 9.4 (SAS Institute, Cary, NC). Time series data of the total number of monthly visits were generated for each participant. Summary statistics, including means and standard deviations, were calculated for each month and across the year for both the pre-period and study-period. Interrupted time series models were used to compare the level (intercept) and changes over time (slope) in visits between the pre-period and study-period. Testing differences in intercepts and slopes indicates whether there are significant changes over time.

To account for within-subject correlation, we used a generalized estimating equations (GEE) model with a Poisson distribution to model monthly visit counts. The estimated monthly visit numbers, along with 95% CIs, were presented to visualize the model-estimated trend for the 2 periods. Due to the sparse number of visits for subtypes, we used binary indicators (Yes vs No) for the monthly visits. The GEE model with a binomial distribution was then used, along with the interrupted time series model, to display predicted probabilities of monthly visits with 95% CIs. To compare the number of visits in a year between the 2 periods, the Wilcoxon signed-rank test was used. All tests were 2-sided, with a significance level of *P* < .05.

## Results

A total of 26 participants were enrolled in the control arm of the study. Baseline demographic and clinical information is reported in Table 1. The average age was 52.9 years (SD = 7.4). The group included 16 females (61.5%) and 10 males (38.5%). Most participants were employed, primarily in domestic (34.6%) and manual labor (23.1%) occupations. Thirteen participants (43.3%) had hypertension, and 1 participant (3.9%) had chronic kidney disease. None had a history of coronary artery disease or cerebrovascular accident. The mean HbA1c was 7.65% (SD = 1.83), and the average duration of diabetes was 6.9 years (SD = 6.0). Of the 26 participants, 24 (92.4%) received oral glucose-lowering medications only.

**Table 1.** Baseline Demographics and Clinical Information for the Participants (n = 26).

Variable	n (%)
Sex	
Female	16 (61.5)
Male	10 (38.5)
Employment	
Domestic	9 (34.6)
Manual labor	6 (23.1)
Food service	3 (11.5)
Unemployed	4 (15.4)
Other/unknown	4 (15.4)
Past Medical History	
Hypertension	13 (43.3)
Coronary artery disease	0 (0.0)
Cerebrovascular accident	0 (0.0)
Chronic kidney disease	1 (3.9)
Diabetes Therapy	
Lifestyle only	1 (3.8)
Oral glucose-lowering medications only	24 (92.4)
Injectables only	0 (0.0)
Oral glucose-lowering medications and injectables	1 (3.8)
Variable	mean (±SD)
Age (years)	52.9 (7.4)
Diabetes diagnosis (years)	6.9 (6.0)
Hemoglobin A1c (%)	7.7 (1.8)
Cholesterol	
Total cholesterol (mg/dL)	178.3 (43.4)
HDL cholesterol (mg/dL)	38.9 (10.3)
LDL cholesterol (mg/dL)	106.0 (32.9)
Triglycerides (mg/dL)	157.4 (131.0)
Blood pressure	
Systolic blood pressure (mmHg)	129.4 (13.8)
Diastolic blood pressure (mmHg)	80.1 (8.1)
Body mass index (kg/m^2^)	31.7 (4.8)
Weight (kg)	82.1 (12.9)

Mean laboratory values were as follows: total cholesterol, 178.3 mg/dL (SD = 43.4); HDL cholesterol, 38.9 mg/dL (SD = 10.3); LDL cholesterol, 106.0 mg/dL (SD = 32.9); and triglycerides, 157.4 mg/dL (SD = 131.0). Mean systolic blood pressure was 129.4 mmHg (SD = 13.8), and mean diastolic blood pressure was 80.1 mmHg (SD = 8.1). The average BMI was 31.7 kg/m^2^ (SD = 4.8), and the mean weight was 82.1 kg (SD = 12.9).

**Figure 1.**
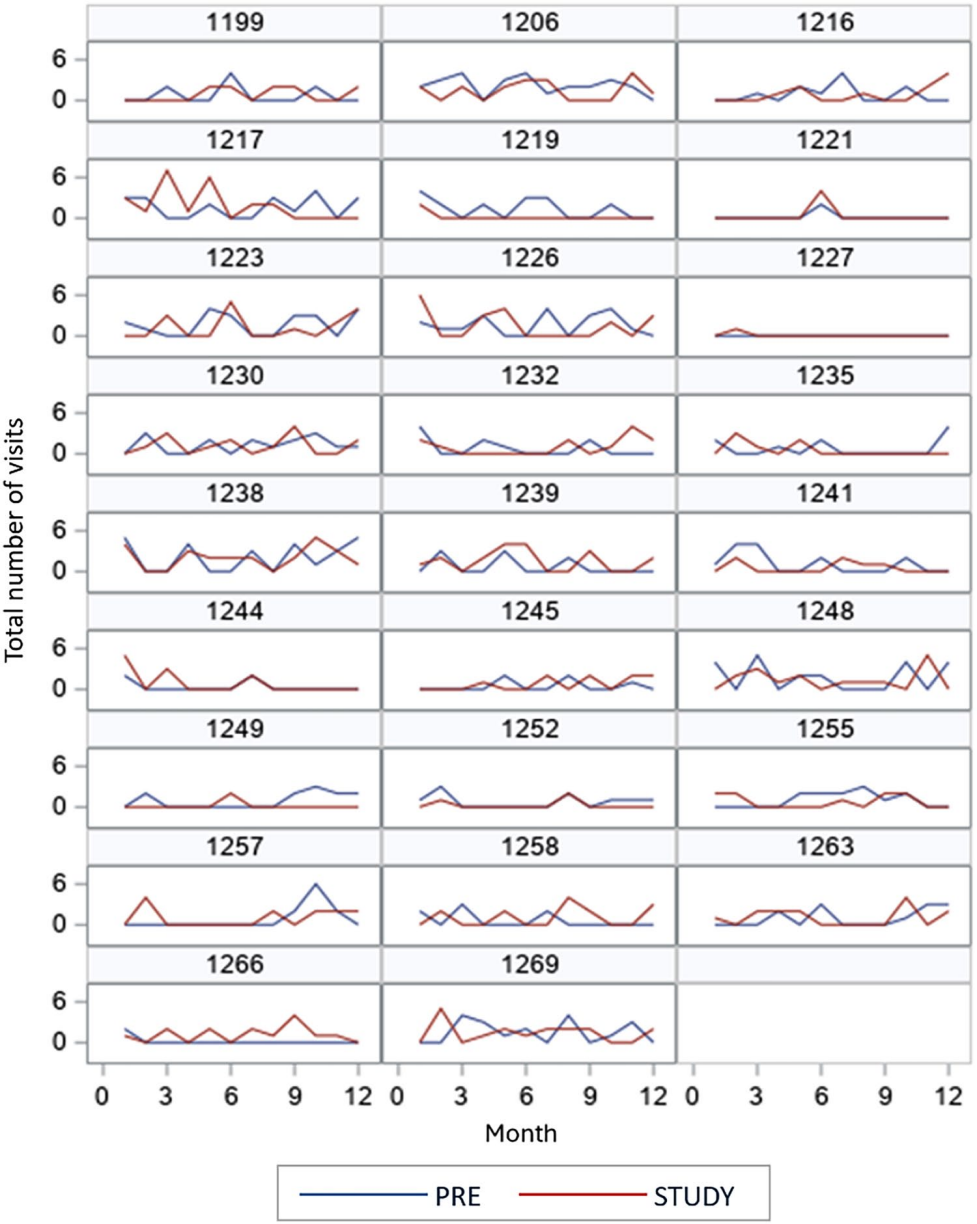
Individual monthly total visits by period. Pre-period: 12-month period prior to study entry. Study-period: 12-month period of the study.

Figure 1 presents the monthly visit counts per study participant during the pre-period and study-period. Mean monthly visits ranged from 0.65 to 1.69 visits/month in the pre-period and from 0.58 to 1.35 visits/month in the study-period. The mean change in monthly visits from the pre- to the study-period ranged from −1.04 to 0.42. The Supplemental Table provides a monthly breakdown of total visits for all participants, comparing the pre-period to the study-period.

**Figure 2.**
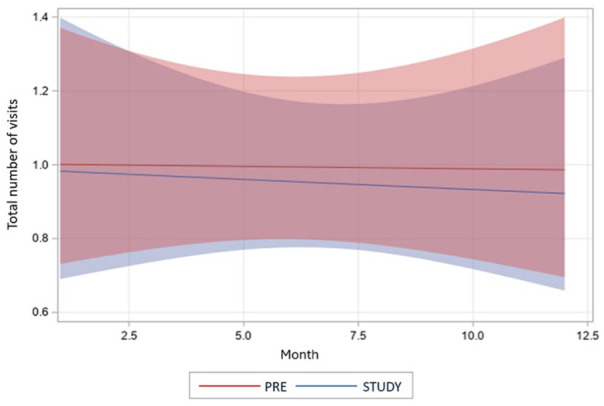
Interrupted time series model depicting the fitted total monthly visits for pre-period and study-period groups (pre: months 1-12, study: months 13-24). Pre-period: 12-month period prior to study entry. Study-period: 12-month period of the study.

Figure 2 displays the estimated number of monthly visits with 95% CIs from the interrupted time series model. There was no significant difference in the fitted mean number of visits in the first month of each period (pre/study-period ratio = 0.98; 95% CI = 0.66, 1.46; *P* = .93). There was no significant difference in changes during the 12 months for the pre-period and study-period, as both curves remained flat with a monthly increment ratio close to 1. The slope comparison also showed no significant difference (pre-/study-period slope ratio = 1.00; 95% CI = 0.93, 1.06; *P* = .89). These results indicate that there was no significant difference in trends, either at baseline or in monthly changes, between the 2 periods.

**Figure 3.**
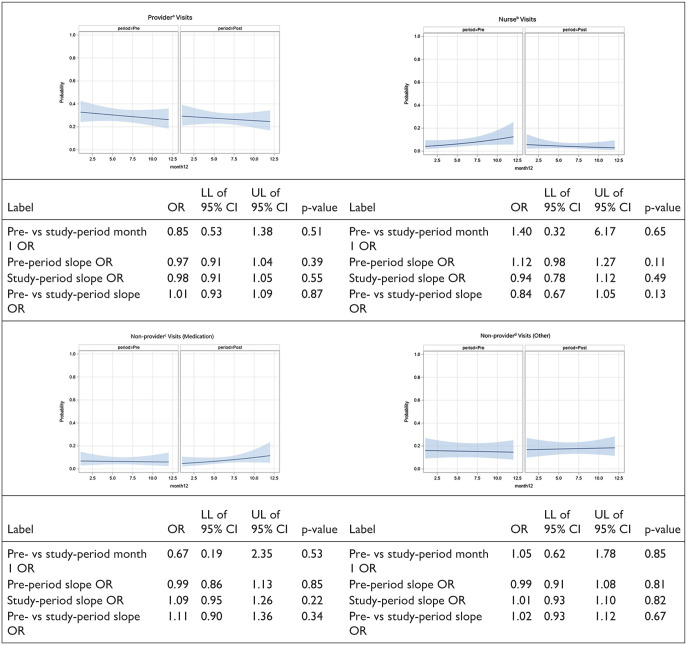
Interrupted time series model for binary outcomes, demonstrating the predicted probabilities by month (pre-period: months 1-12, study-period: months 13-24) for provider, nurse, and non-provider visits with 95% confidence intervals (CIs). ^a^**Provider:** MD, DO, Advance Practice Provider. ^b^**Nurse:** Vital signs (eg, blood pressure measurements). ^c^**Non-provider (medication):** Medication refills. ^d^**Non-provider (other):** Lab orders, eligibility paperwork.

Figure 3 presents the results of an interrupted time series model, describing the predicted probabilities by month for provider, nurse, non-provider (medication), and non-provider (other) visits. There were no significant differences in the predicted probability of a visit at month 1 between the pre-period and the study-period for provider visits (OR = 0.85; 95% CI = 0.53, 1.38), nurse visits (OR = 1.40; 95% CI = 0.32, 6.17), non-provider (medication) visits (OR = 0.67; 95% CI = 0.19, 2.35), or non-provider (other) visits (OR = 1.05; 95% CI = 0.62, 1.78). Additionally, the slope ORs for both periods showed no significant trends in the probability of visits over time for any visit type. There were no significant differences in the slope OR for provider visits (OR = 1.01; 95% CI = 0.93, 1.09), nurse visits (OR = 0.84; 95% CI = 0.67, 1.05), non-provider (medication) visits (OR = 1.11; 95% CI = 0.90, 1.36), or non-provider (other) visits (OR = 1.02; 95% CI = 0.93, 1.12).

Table 2 summarizes yearly totals for all visit types in the pre-period and study-period. Median total yearly visits increased from 10.5 (IQR = 8, 18) in the pre-period to 12 (interquartile range [IQR] = 6, 16) in the study-period, though the median change was −0.5 visits (IQR = −5, 3). For provider, nurse, and non-provider (medication) visits, the median annual change was 0: provider: 0 (IQR = −2, 1); nurse: 0 (IQR = −1, 0); non-provider (medication): 0 (IQR = 0, 1). Non-provider (other) visits showed a slight increase, with a median change in annual visits of 0.5 (IQR = −1, 2).

**Table 2.** Yearly Summations for Total, Provider, Nurse, Non-provider (Other), and Non-provider (Medication) Visits.

Variable	Mean	Std	Min	P25	Median	P75	Max
Total visits (pre)	11.92	6.99	0	8	10.5	18	26
Total visits (study)	11.42	6.2	1	6	12	16	24
Total visits (change)	−0.5	5.91	−14	−5	−0.5	3	12
Provider visits (pre)	3.92	2.02	0	3	4	5	8
Provider visits (study)	3.46	1.7	1	2	3	5	6
Provider visits (change)	−0.46	2.04	−6	−2	0	1	3
Nurse visits (pre)	0.96	1.22	0	0	1	1	4
Nurse visits (study)	0.5	0.71	0	0	0	1	3
Nurse visits (change)	−0.46	1.39	−4	−1	0	0	2
Non-provider (medication) visits (pre)	0.81	1.06	0	0	0.5	1	4
Non-provider (medication) visits (study)	0.96	0.92	0	0	1	2	3
Non-provider (medication) visits (change)	0.15	0.83	−2	0	0	1	1
Non-provider (other) visits (pre)	2.27	2.39	0	0	1.5	5	8
Non-provider (other) visits (study)	2.88	2.6	0	1	2	4	10
Non-provider (other) visits (change)	0.62	2.35	−4	−1	0.5	2	7

## Discussion

We conducted a secondary exploratory analysis of healthcare utilization among control participants in a 12-month RCT conducted in a low-income setting. Based on prior literature on the Hawthorne effect,^2,12^ we hypothesized that control participants would increase healthcare utilization during the study compared to the year prior, due to their awareness of study involvement and receipt of clinical information. However, this hypothesis was not supported; no significant differences in utilization were observed. Despite gaining knowledge of their clinical values, control participants did not alter their healthcare utilization. This finding is important for investigators working with low-income populations, where concerns may arise that increased clinical contact or access in the control arm could inflate healthcare utilization and mask true intervention effects.

Several explanations could account for the lack of behavioral changes observed in the control arm. One possibility is that participants did not fully understand their clinical values and therefore did not find it necessary to follow-up. While this is plausible, it is important to note that values were entered into the electronic medical record for provider follow-up, and urgent values were addressed immediately. Another possibility is that the Hawthorne effect may not be as potent as initially suggested. A systematic review of 19 studies revealed some evidence of the effect; however, the high degree of study heterogeneity made it challenging to determine the degree or conditions under which it occurred, and observed alterations within the study setup could have been influenced by factors such as the novelty of the study or various forms of bias.^12^

Furthermore, it is possible that manifestations of the Hawthorne effect differ in low-income settings. Healthcare in these settings differs significantly from that in higher-income populations due to limited access to care, reduced ability to implement lifestyle and other changes (eg, modifications in medications or diet) in the context of restricted income, low health literacy, transportation barriers, and various social and economic determinants of health.^17,18^ After receiving their baseline measurements, control participants may have been unable to schedule follow-up appointments due to limitations in clinic infrastructure, lack of internet access, inflexible work schedules, and associated costs. In higher-income settings, individuals who learn of a poor clinical outcome are more likely to secure follow-up appointments, given the lower likelihood of encountering these barriers. Thus, access to care may remain the primary obstacle to healthcare for low-income individuals, rather than mere knowledge of their health conditions.

### Strengths, Limitations, and Future Directions

The study contributes valuable data on healthcare behaviors in the control arm within a low-income setting, a population often underrepresented in clinical trials despite being disproportionately affected by chronic diseases.^19^ By examining outcomes in the absence of an active intervention, the study provides important context for understanding baseline utilization patterns in low-income settings. The transparent discussion of null findings further underscores how structural barriers, such as limited access to care and competing socioeconomic demands, may mitigate the behavioral effects of study participation. This perspective highlights the critical role of contextual factors in shaping intervention outcomes and adds to the growing literature on health disparities and implementation science.

The study findings are limited by their exploratory nature and the small sample size of Hispanic participants from a single site, which may reduce generalizability and the ability to detect subtle behavioral changes. Several potential confounding factors may have influenced the observed patterns of healthcare utilization, independent of the intervention itself. For instance, individuals with higher baseline HbA1c levels or more complex medical needs may naturally require more frequent clinic visits, regardless of study participation. Similarly, variations in health literacy could affect both participants’ understanding of their health status and their likelihood of engaging in behavior change, potentially influencing utilization outcomes. Unmeasured comorbidities may also contribute to increased healthcare needs, while external factors, such as seasonal fluctuations in clinic operations or concurrent community health initiatives, could have impacted visit frequency. Recognizing these potential confounders is important for interpreting the findings and highlights the challenges of evaluating behavioral interventions in real-world, low-income settings.

The study findings highlight the need for future mixed-methods studies that combine qualitative interviews with quantitative data, particularly in households classified as ALICE (Asset Limited, Income Constrained, and Employed).^20,21^ Such research could better identify actionable barriers to optimal diabetes care in resource-restricted communities, ultimately informing policy and practice.

## Conclusions

This study explored behavioral changes among control participants in low-income settings, testing the hypothesis that awareness of baseline health measurements would increase healthcare utilization in the control arm. This would have supported the Hawthorne effect, but no such effect was observed. This finding may reflect the structural and socioeconomic barriers faced by individuals in low-income communities, which can limit their ability to translate awareness into action. Although limited by small sample size, these exploratory findings offer preliminary insights into how structural barriers, more than patient knowledge, may influence healthcare behaviors in low-income populations, underscoring critical considerations for future interventions.

## Supplemental Material

sj-docx-1-jpc-10.1177_21501319251379740 – Supplemental material for Healthcare Utilization Unchanged in the Control Arm of a Randomized Clinical Trial Supplemental material, sj-docx-1-jpc-10.1177_21501319251379740 for Healthcare Utilization Unchanged in the Control Arm of a Randomized Clinical Trial by Pratik Gongloor, Saad Nadeem, Xiaoying Yu, Mukaila Raji, Kristina D. Mena and Elizabeth M. Vaughan in Journal of Primary Care & Community Health

## References

[1] Best M. Walter A Shewhart, 1924, and the Hawthorne factory. Qual Saf Health Care. 2006;15(2):142-143. doi:10.1136/qshc.2006.018093 16585117 PMC2464836

[2] McCambridge J Witton J Elbourne DR. Systematic review of the Hawthorne effect: new concepts are needed to study research participation effects. J Clin Epidemiol. 2014;67(3):267-277. doi:10.1016/j.jclinepi.2013.08.015 24275499 PMC3969247

[3] Purssell E Drey N Chudleigh J Creedon S Gould DJ. The Hawthorne effect on adherence to hand hygiene in patient care. J Hosp Infect. 2020;106(2):311-317. doi:10.1016/j.jhin.2020.07.028 32763330

[bibr4-21501319251379740] Rosenberg M Pettifor A Twine R , et al Evidence for sample selection effect and Hawthorne effect in behavioural HIV prevention trial among young women in a rural South African community. BMJ Open. 2018;8(1):e019167. doi:10.1136/bmjopen-2017-019167 PMC5781067 29326192

[bibr5-21501319251379740] Goodwin MA Stange KC Zyzanski SJ Crabtree BF Borawski EA Flocke SA. The Hawthorne effect in direct observation research with physicians and patients. Eval Clin Pract. 2017;23(6):1322-1328. doi:10.1111/jep.12781 PMC5741487 28752911

[6] McCarney R Warner J Iliffe S Van Haselen R Griffin M Fisher P. The Hawthorne effect: a randomised, controlled trial. BMC Med Res Methodol. 2007;7(1):30. doi:10.1186/1471-2288-7-30 17608932 PMC1936999

[bibr7-21501319251379740] Lubman DI Grigg J Reynolds J , et al Effectiveness of a stand-alone telephone-delivered intervention for reducing problem alcohol use: a randomized clinical trial. JAMA Psychiatry. 2022;79(11):1055. doi:10.1001/jamapsychiatry.2022.2779 36129698 PMC9494267

[bibr8-21501319251379740] Berthelot JM Nizard J Maugars Y. The negative Hawthorne effect: explaining pain overexpression. Joint Bone Spine. 2019;86(4):445-449. doi:10.1016/j.jbspin.2018.10.003 30316973

[9] Wolfe F Michaud K. The Hawthorne effect, sponsored trials, and the overestimation of treatment effectiveness. J Rheumatol. 2010;37(11):2216-2220. doi:10.3899/jrheum.100497 20843902

[10] Kompier MA. The “Hawthorne effect” is a myth, but what keeps the story going? Scand J Work Environ Health. 2006;32(5):402-412. doi:10.5271/sjweh.1036 17091208

[11] Wu KS Lee SSJ Chen JK , et al Identifying heterogeneity in the Hawthorne effect on hand hygiene observation: a cohort study of overtly and covertly observed results. BMC Infect Dis. 2018;18(1):369. doi:10.1186/s12879-018-3292-5 30081843 PMC6090841

[12] Berkhout C Berbra O Favre J , et al Defining and evaluating the Hawthorne effect in primary care, a systematic review and meta-analysis. Front Med. 2022;9:1033486. doi:10.3389/fmed.2022.1033486 PMC9679018 36425097

[13] Escobedo LE Cervantes L Havranek E. Barriers in healthcare for Latinx patients with limited english proficiency—a narrative review. J Gen intern Med. 2023;38(5):1264-1271. doi:10.1007/s11606-022-07995-3 36720766 PMC9888733

[bibr14-21501319251379740] Velasco-Mondragon E Jimenez A Palladino-Davis AG Davis D Escamilla-Cejudo JA. Hispanic health in the USA: a scoping review of the literature. Public Health Rev. 2016;37(1):31. doi:10.1186/s40985-016-0043-2 29450072 PMC5809877

[15] Escarce JJ Kapur K . Access to and quality of health care. In: Tienda M Mitchell F (eds) National Research Council (US) Panel on Hispanics in the United States. Chapter 10. National Academies Press (US); 2006: 410-447.

[16] Vaughan EM Yu X Cardenas VJ , et al From trials to practice: implementing a clinical intervention in community settings. J Prim Care Community Health. 2025;16:21501319251339190. doi:10.1177/21501319251339190 PMC12106995 40418742

[17] Wolfe MK McDonald NC Holmes GM. Transportation barriers to health care in the United States: findings from the national health interview survey, 1997–2017. Am J Public Health. 2020;110(6):815-822. doi:10.2105/AJPH.2020.305579 32298170 PMC7204444

[18] Hu J Amirehsani K Wallace DC Letvak S. Perceptions of barriers in managing diabetes: perspectives of hispanic immigrant patients and family members. Diabetes Educ. 2013;39(4):494-503. doi:10.1177/0145721713486200 23640301 PMC4054933

[19] Li G Zhang J Van Spall HGC , et al Exploring ethnic representativeness in diabetes clinical trial enrolment from 2000 to 2020: a chronological survey. Diabetologia. 2022;65(9):1461-1472. doi:10.1007/s00125-022-05736-z 35705796 PMC9200441

[20] National Overview | UnitedForALICE. 2025. Accessed August 29, 2025. https://www.unitedforalice.org/national-overview

[21] Overview | UnitedForALICE. 2009-2025 United Way of Northern New Jersey. Accessed August 29, 2025. https://www.unitedforalice.org/consequences

