# Supplementary material for: Healthcare Utilization Unchanged in the Control Arm of a Randomized Clinical Trial
Source: J Prim Care Community Health. 2025 Sep 27;16:21501319251379740. doi: 10.1177/21501319251379740 (PMC12476492; doi:10.1177/21501319251379740)
Supplement: sj-docx-1-jpc-10.1177_21501319251379740 – Supplemental material for Healthcare Utilization Unchanged in the Control Arm of a Randomized Clinical Trial [file sj-docx-1-jpc-10.1177_21501319251379740.docx]

| Supplemental Table. Total visits for all participants by month (n=26), where *pre-period* represents the year before the study (months 1-12) and *study-period* represents the year of the study (months 13-24) | | | | | | |
| --- | --- | --- | --- | --- | --- | --- |
| Month | **Variable** | **Mean** | **Std Dev** | **Minimum** | **Median** | **Maximum** |
| 1, 13 | pre-period | 1.38 | 1.58 | 0 | 1 | 5 |
|  | study-period | 1.12 | 1.7 | 0 | 0 | 6 |
|  | change | -0.27 | 1.66 | -4 | 0 | 4 |
| 2, 14 | pre-period | 0.96 | 1.37 | 0 | 0 | 4 |
|  | study-period | 1.04 | 1.37 | 0 | 0.5 | 5 |
|  | change | 0.08 | 2 | -3 | 0 | 5 |
| 3, 15 | pre-period | 0.92 | 1.62 | 0 | 0 | 5 |
|  | study-period | 1 | 1.7 | 0 | 0 | 7 |
|  | change | 0.08 | 2.37 | -4 | 0 | 7 |
| 4, 16 | pre-period | 0.65 | 1.2 | 0 | 0 | 4 |
|  | study-period | 0.58 | 0.95 | 0 | 0 | 3 |
|  | change | -0.08 | 0.93 | -2 | 0 | 2 |
| 5, 17 | pre-period | 0.92 | 1.23 | 0 | 0 | 4 |
|  | study-period | 1.35 | 1.57 | 0 | 1.5 | 6 |
|  | change | 0.42 | 1.79 | -4 | 0 | 4 |
| 6, 18 | pre-period | 1.15 | 1.41 | 0 | 0 | 4 |
|  | study-period | 0.96 | 1.54 | 0 | 0 | 5 |
|  | change | -0.19 | 1.74 | -3 | 0 | 4 |
| 7, 19 | pre-period | 0.88 | 1.37 | 0 | 0 | 4 |
|  | study-period | 0.73 | 1 | 0 | 0 | 3 |
|  | change | -0.15 | 1.74 | -4 | 0 | 2 |
| 8, 20 | pre-period | 0.73 | 1.22 | 0 | 0 | 4 |
|  | study-period | 0.81 | 1.06 | 0 | 0 | 4 |
|  | change | 0.08 | 1.52 | -3 | 0 | 4 |
| 9, 21 | pre-period | 0.85 | 1.22 | 0 | 0 | 4 |
|  | study-period | 1 | 1.3 | 0 | 0 | 4 |
|  | change | 0.15 | 1.8 | -3 | 0 | 4 |
| 10, 22 | pre-period | 1.69 | 1.67 | 0 | 1.5 | 6 |
|  | study-period | 0.65 | 1.32 | 0 | 0 | 5 |
|  | change | -1.04 | 2.05 | -4 | -1 | 4 |
| 11, 23 | pre-period | 0.73 | 1.08 | 0 | 0 | 3 |
|  | study-period | 0.96 | 1.54 | 0 | 0 | 5 |
|  | change | 0.23 | 1.77 | -3 | 0 | 5 |
| 12, 24 | pre-period | 1.04 | 1.66 | 0 | 0 | 5 |
|  | study-period | 1.23 | 1.34 | 0 | 1 | 4 |
|  | change | 0.19 | 2.21 | -4 | 0 | 4 |
